# Blood-Borne ST6GAL1 Regulates Immunoglobulin Production in B Cells

**DOI:** 10.3389/fimmu.2020.00617

**Published:** 2020-04-23

**Authors:** Eric E. Irons, Patrick R. Punch, Joseph T. Y. Lau

**Affiliations:** Department of Molecular and Cellular Biology, Roswell Park Comprehensive Cancer Center, University at Buffalo, Buffalo, NY, United States

**Keywords:** sialic acid, sialyltransferase, B cell, IgG, regulation – post-transcriptional, ST6Gal I, extracellular, circulating factor

## Abstract

Humoral immunity is an effective but metabolically expensive defense mechanism. It is unclear whether systemic cues exist to communicate the dynamic need for antigen presentation and immunoglobulin production. Here, we report a novel role for the liver-produced, acute phase reactant ST6GAL1 in IgG production. B cell expression of ST6GAL1, a sialyltransferase mediating the attachment of α2,6-linked sialic acids on N-glycans, is classically implicated in the dysregulated B cell development and immunoglobulin levels of *St6gal1*-deficient mice. However, the blood-borne pool of ST6GAL1, upregulated during systemic inflammation, can also extrinsically modify leukocyte cell surfaces. We show that B cell independent, extracellular ST6GAL1 enhances B cell IgG production and increases blood IgG titers. B cells of mice lacking the hepatocyte specific *St6gal1* promoter have reduced sialylation of cell surface CD22 and CD45 and produce less IgG upon stimulation. Sialylation of B cells by extracellular ST6GAL1 boosts expression of IgM, IgD, and CD86, proliferation, and IgG production *in vitro*. *In vivo*, elevation of blood ST6GAL1 enhances B cell development and systemic IgG in a CD22-dependent manner. Our data point to a function of an extracellular glycosyltransferase in promoting humoral immunity. Manipulation of systemic ST6GAL1 may represent an effective therapeutic approach for humoral insufficiency.

## eTOC Summary

The expression of the sialyltransferase ST6GAL1 is essential to the development of a robust and tolerant humoral immunity. We report a novel role for the liver-produced, acute phase protein ST6GAL1 in enhancing B cell development and in IgG production in a CD22-dependent manner *in vivo*.

## Introduction

Acute phase reactants are widely used as biomarkers of rheumatic and inflammatory diseases (1, 2). Although these circulating factors are generally thought to restore homeostasis during systemic inflammation, a complete understanding of their functions in normal immune responses is still lacking. ST6GAL1, a sialyltransferase mediating the addition of α2,6-linked sialic acid to N-glycans, has long been recognized as an acute phase reactant, with levels in the blood peaking 96 h after an infectious or traumatic insult (3–6). Associations have previously been reported between plasma glycoprotein sialylation and inflammatory states, as well as between ST6GAL1 polymorphisms and autoimmune diseases (7–10). However, an understanding of how extracellular ST6GAL1 functions in immunity has remained elusive due to the simultaneous expression of intracellular ST6GAL1 within the leukocytes and their precursors. Recent studies suggest an unexpected role for blood ST6GAL1 in the direct enzymatic modification of cell-surface and soluble glycoproteins (11–13). Extracellular ST6GAL1 has been shown to directly sialylate bone marrow granulocyte/monocyte progenitors (GMP) to suppress G-CSF induced granulocyte differentiation (14–17). Serum ST6GAL1 is also implicated in the sialylation of IgG, enabling binding to inhibitory FcγRIIb, the primary mechanism by which intravenous immunoglobulin (IVIG) alleviates autoimmune disease (18–20). By restricting myeloid cell production and activation, extrinsic sialylation by ST6GAL1 is an emerging regulator of both classical and allergic inflammation (6, 21, 22).

The expression of ST6GAL1 is essential to the development of a robust and tolerant humoral immune system (21–23). Sialylation by ST6GAL1 disrupts the engagement of ITIM-containing inhibitory lectins CD22 and Siglec-G, altering their localization relative to the B cell receptor (BCR) and lipid rafts in the plasma membrane (24, 25). By sequestration of CD22 away from the BCR, sialic acid in *cis* potentiates antigen-induced activation, marginal zone B cell development, as well as T-dependent and T-independent immune responses (25–28). Conversely, engagement of CD22 by sialic acid in *trans* recruits CD22 to the immune synapse, enforcing tolerance to self-antigens (24, 29). It has long been presumed that the synthesis of sialylated CD22 ligands is a direct result of the action of cell-intrinsic sialyltransferases within the ER-Golgi complex. However, recent observations demonstrate a role for host-derived, cell non-autonomous ST6GAL1 in the sialylation and survival of immature B cell populations, calling this canonical model into question (30). The functional consequences of circulatory ST6GAL1 on humoral immunity, if any, remain unknown.

Here we report a role for extracellular ST6GAL1 in modulating IgG production. Wild-type B cells reconstituted in ST6GAL1-deficient hosts exhibited compromised IgG production, resulting in diminished *in vivo* total and antigen-specific IgG. Conditional ablation of *St6gal1* in hepatocytes resulted in diminished sialylation of B cell CD22 and CD45 and reproduced the attenuated IgG production upon stimulation. Sialylation of immature B cells boosted BCR-induced proliferation and IgG secretion *ex vivo*. Elevation of blood ST6GAL1 *in vivo* was able to enhance follicular B cell development and increase total blood IgG in a CD22-dependent manner. Our findings demonstrate a novel, hepatic axis of extrinsic sialylation by ST6GAL1 that mediates B cell functions via CD22, with possible therapeutic implications for the treatment of humoral immunodeficiency.

## Materials and Methods

### Animal Models and Bone Marrow Transplantation

C57BL/6J (WT) and B6.μMT mice were purchased from Jackson Laboratory. *St6gal1*-KO and dP1 mice have been previously described and were backcrossed onto a C57BL/6J background for at least 15 generations (23, 31). μMT/*St6gal1*-KO mice were generated by crossing single-knockouts, as described previously (30). CD22-KO mice were purchased from Jackson Laboratory. For bone marrow transplantation, recipient mice were subjected to full body gamma-irradiation (600 rads), then rescued with 4.0 × 10^6^ total bone marrow cells of indicated donor genotype(s). Blood was collected from the retro-orbital venous sinus at indicated time points post-BMT for serum analysis and cell sorting. Unless otherwise stated, mice between 6 and 12 weeks of age were used for experiments. Roswell Park Institutional Animal Care and Use Committee approved maintenance of animals and all procedures used under protocol 1071M.

### Antibodies

For flow cytometry, anti-CD45.1-PerCP/Cy5.5 (A20), anti-CD45.2-FITC (104), anti-B220-PE/Cy7 (RA3-6B2), anti-CD19-BV510 (GD5), anti-IgD-PE (11-26c.2a), anti-CD23-APC/Cy7 (B3B4), anti-IgM-APC (RMM-1), anti-CD21-BV421 (7E9), anti-CD24-PE (30-F1), anti-CD138-BV421 (281-2), anti-BP-1-PerCP/Cy5.5 (6C3), anti-CD93-PerCP/Cy5.5 (AA4.1), anti-CD86-APC/Cy7 (GL-1), anti-IgG1-FITC (RMG1-1), anti-IgG2a-APC (RMG2a-62), anti-IgG2b-PE (RMG2b-1), anti-IgG3-biotin (RMG3-1) were purchased from BioLegend. SNA-FITC was purchased from Vector Laboratories. Anti-pZap-70 (Y319)/pSyk (Y352)-AlexaFluor488 (65E4) was purchased from Cell Signaling Technology. For magnetic cell separation, biotinylated anti-IgM (RMM-1), anti-B220 (RA3-6B2), and anti-Gr1 (RB6-8C5), were purchased from BD Pharmingen, and anti-IgD-PE (11-26c.2a) from Biolegend. Streptavidin-conjugated microbeads or anti-PE microbeads (Miltenyi Biotechnology) were used to pull down labeled cells. For microscopy, anti-IgM-Cy3 (EMD Millipore), anti-IgD-FITC (11-26c, Invitrogen), anti-B220 (RA3-6B2, eBioscience), and PNA-Fluorescein lectin (Vector Biolabs) were used. For western blotting, anti-β-actin (BA3R, Invitrogen), anti-ST6GAL1 (R&D Biosystems), anti-CD22 (308501, Thermo Fisher), anti-B220/CD45-biotin (RA3-6B2, BD Biosciences), anti-IgM-biotin (RMM-1, Biolegend), anti-rat-HRP (C60992, LS Bio), streptavidin-HRP (N100, Thermo Fisher), anti-goat-HRP (HAF109, R&D Biosystems), and goat anti-mouse-HRP (Bio-Rad).

### Immunoglobulin Quantification

IgG was quantified in serum and *in vitro* conditioned supernatant by ELISA (Bethyl Laboratories). For total IgG, serum samples were diluted between 1:5000 and 1:50,000, and conditioned supernatant diluted 1:3. Standards of mouse serum were included in duplicate for quantification of unknowns. For antigen-specific IgG, plates were coated overnight with 10 μg/ml NP-Ova before blocking. IgG titers were quantified by comparison with standards in anti-IgG coated wells in parallel. Absorbance (650 nm) was quantified using a Synergy HTX Reader (Biotek). For analysis, standard values were modeled by best-fit equations (linear or logarithmic), which were used to infer concentrations of unknowns.

### Flow Cytometry

Bone marrow cells were collected by flushing femurs, splenocytes by dissociating and filtering spleens, and peripheral blood collected in citrate-based anticoagulant. All tissues were subjected to ACK lysis to remove anucleated cells. Cells were stained in flow cytometry buffer (1 mM EDTA, 0.02% sodium azide, 0.05% BSA in PBS) with indicated antibodies at 1:100-1:200 dilution. For intracellular p-Syk staining, B cells were stimulated for indicated times, then fixed in 5% formalin for 10 min, washed, and resuspended in BD Cytoperm buffer (BD Biosciences) for 20 min. Cells were then incubated in BD Cytoperm buffer with anti-pSyk antibody (1:100) for 30 min, washed, and analyzed by flow cytometry. All flow cytometry data was collected on BD LSRII cytometer and analyzed with FlowJo software.

### *In vivo* IgG Half-life Determination

Hundred μg of Chrompure mouse polyclonal IgG (Jackson ImmunoResearch) was injected intraperitoneally into μMT or μMT/*St6gal1*-KO mice, and serial blood samples collected between 1 and 9 days after administration. IgG concentrations, quantitated by ELISA, were plotted and fitted to exponential equations, which were used to determine the half-life of IgG.

### *Ex vivo* IgG Production

Mature B cells (IgD+/IgM-low) from peripheral blood of BMT chimeras at 4–6 weeks post-transplant were sorted by fluorescence-activated cell sorting using BD FacsAria cytometer. Purity was routinely 95%+. In other experiments, bone marrow IgD+ mature B cells from WT or *dP1* mice were isolated by magnetic separation (MACS column, Miltenyi Biotechnology). Enriched cells were washed and enumerated, then activated by functional grade mouse anti-IgM (eBioscience), anti-CD40 (eBioscience, HM40-3) and either IL-4 (100 ng/ml, Gibco) or LPS (25 μg/ml) for 3 or 6 days in complete culture medium at 37°C. Cells were then centrifuged at 1000 rpm and cell-free supernatant collected and stored at −80°C until further analysis.

### RNA Analysis

*Ex vivo* cultured cells were pelleted, then resuspended in TRI reagent (MRC Inc.) and frozen at −80°C. RNA was extracted under RNAse-free conditions according to manufacturer guidelines, then quantified and all samples normalized prior to cDNA synthesis of 750–2000 ng of RNA by iScript cDNA Synthesis kit (Bio-Rad). cDNA was amplified by previously described primers using iQ SYBR Green kit (Bio-Rad) (32). All transcripts were normalized to control genes (IgB or β-actin), then relative expression levels normalized to the biological control (WT or mock-treated cells) to obtain fold-change values.

### Co-immunoprecipitation

Equivalent numbers of IgD+ bone marrow mature B cells were subjected to membrane protein extraction using Mem-PER Plus kit (Thermo Fisher Scientific). Protein concentration was quantitated by BCA protein assay test (Thermo Fisher Scientific) and normalized prior to overnight incubation with 100 μl of pre-blocked SNA-agarose or agarose beads (Vector Laboratories). Unbound supernatant was saved, and beads were extensively washed before bound protein was boiled off in 50 μl Laemmli buffer with 2-mercaptoethanol. Samples were resolved in a 4–15% gradient gel (Bio-Rad), then transferred to methanol-activated PVDF membranes, blocked for 1 h in 3% BSA solution, and incubated with primary antibodies overnight. Membranes were washed, incubated in secondary antibody for 1 h, then developed with Pierce ECL WB Substrate (Thermo Fisher Scientific) and immediately imaged using ChemiDoc Touch (Bio-Rad).

### *Ex vivo* Immature B Cell Maturation

Developing B cells were isolated from bone marrow as described previously (30). Briefly, total bone marrow cells were depleted for IgM+, IgD+, and Gr-1+ cells, then positively selected for B220 + cells by MACS column (Miltenyi Biotechnology). Resulting cells were treated with 33 ng/ml recombinant murine BAFF (R&D Biosystems), 0.05 mM CMP-sialic acid, and either 10 μg/ml rat ST6GAL1 or equivalent vehicle control for 3 days. Resulting cultures were analyzed by flow cytometry or washed twice in PBS, then stimulated for IgG production with anti-IgM, anti-CD40, and IL-4 or LPS (25 μg/ml). Cell counts were acquired using Bio-Rad TC20 Automated Cell Counter. Recombinant rat secretory ST6GAL1 was a generous gift from Dr. Kelley Moremen of the University of Georgia.

### *In vivo* rST6G Treatment

Wild-type mice were treated with 60 ng BAFF, 10^–7^ moles CMP-Neu5Ac (Sigma Aldrich), and either 30 μg ST6GAL1 or equivalent volume of mock solution by tail vein injection. Animals were sacrificed 4 days later for analysis of bone marrow and splenic B cells by flow cytometry and microscopy.

### Microscopy

Snap-frozen spleens were sectioned onto charged glass slides, fixed in acetone at −20°C, then rehydrated twice in PBS. Slides were blocked for 1 h in 5% BSA, then stained overnight with anti-IgD-FITC and anti-IgM-Cy3 or anti-B220-biotin and *peanut agglutinin* (PNA)-FITC (Vector Biolabs). Slides were washed and incubated with secondary antibody for 1 h where necessary. Stained slides were mounted in 10% glycerol with glass cover slips, then visualized immediately using a Nikon Eclipse E600 microscope with EXFO X-cite 120 light source. Spot RT3 camera and Spot Software were used to capture images.

### Hydrodynamic Transfection

pLive plasmids (Mirus Bio) containing AFP enhancer and a downstream minimal albumin promoter were purchased. Rat secretory ST6GAL1 DNA sequence (∼600 bp) was inserted between introns 1 and 2 and expressed in transformed *E. coli*. Successful insertion was confirmed by two restriction digests. Mice were intravenously administered 20 μg of empty vector or rST6-containing plasmids in a volume of 2 ml saline. Blood collections were made prior to transfection and at indicated times. Tissue analysis by flow cytometry was performed upon sacrifice. Quantification of sialyltransferase activity in serum was performed using an artificial O-benzyl-linked disaccharide acceptor, as described previously (11).

## Results

### B Cell Independent ST6GAL1 Regulates Humoral Immunity

**FIGURE 1:**
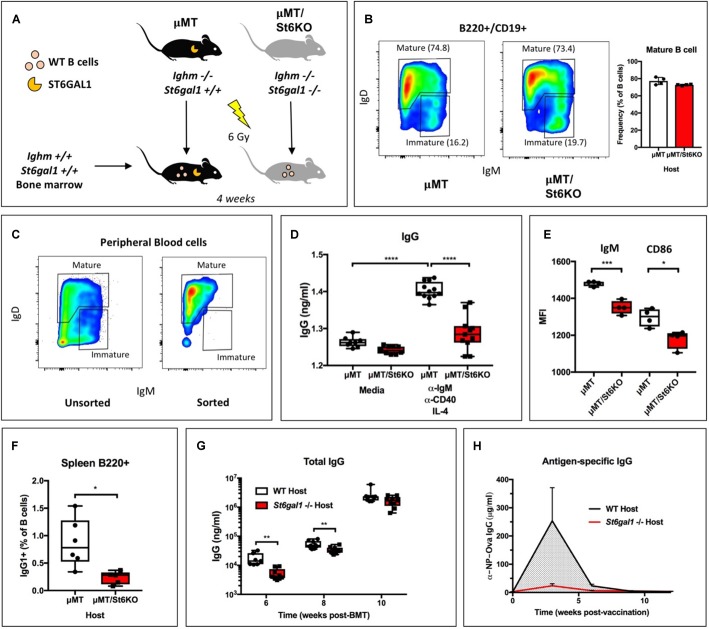
B cell-independent ST6GAL1 potentiates IgG production from mature B cells. **(A)** B cell-deficient (μMT) mice that have or lack *ST6GAL1* were irradiated and reconstituted with wild-type whole bone marrow. **(B)** Blood mature B cells in chimeras at 4 weeks post-transplantation (*n* = 4). **(C)** Blood mature B cells were sorted by FACS and cultured at equal cell number for 6 days to assess antibody production **(D)** IgG in conditioned medium was quantified by ELISA after stimulation (*n* = 8–12) and **(E)** cell surface expression of IgM and CD86 determined by flow cytometry (*n* = 4). **(F)** Splenic IgG1 + B220 + cells in bone marrow chimeras with indicated recipient genotype (*n* = 5–6). **(G)** WT bone marrow was used to reconstitute irradiated WT or *St6gal1–/–* hosts, and total blood IgG was quantitated by ELISA at indicated time points post-transplant (*n* = 7–9). **(H)** Chimeras were immunized with a single dose of NP-Ova in CFA. NP-Ova-specific IgG in the serum was quantified at indicated time points post-vaccination (*n* = 5). *p < 0.05, **p < 0.01, ***p < 0.001, ****p < 0.0001.

Global genetic inactivation of *St6gal1* leads to humoral immunodeficiency, with severely reduced antigen-induced proliferation, antibody production, and marginal zone B cells (23). However, it remains unclear if the importance of ST6GAL1 for B cell development and functions is solely a result of B cell-intrinsic expression of the enzyme. To determine whether B cell-independent ST6GAL1 was relevant to the functions of *St6gal1-*competent B cells, we reconstituted irradiated *St6gal1*+*/*+ or *St6gal1−/−* mice with WT donor bone marrow. Recipient mice also harbored a targeted disruption of the *Ighm* gene (μMT background) and were natively deficient in IgM+ immature and mature B cells, ensuring that all mature B cells were donor-derived and *St6gal1*-competent (Figure 1A) (33). Four weeks post-transplantation, analysis of peripheral blood B cells by flow cytometry revealed no significant difference in the abundance of recirculating mature B cells (B220+/CD19+/IgD+) due to recipient *St6gal1* status (Figure 1B). At 8 weeks post-transplantation, analysis of B cell subpopulations demonstrated similar reconstitution of mature B cells in the bone marrow and spleen (Supplementary Figure S1). To understand whether mature B cells were functionally different due to host-derived ST6GAL1, we purified mature B cells from peripheral blood by FACS and stimulated equivalent numbers of cells at the B cell receptor (anti-IgM), along with co-stimulatory anti-CD40 and IL-4 for 6 days (Figure 1C). Mature B cells that developed in an *St6gal1−*/*−* host environment secreted significantly less IgG, accompanied by reduced expression of the B cell receptor (IgM) and co-stimulatory CD86 (Figures 1D,E). We also noted a striking reduction in class-switched IgG + B cells within the spleens of *St6gal1−/−* hosts (Figure 1F). To test if host ST6GAL1 influenced total blood IgG levels after transplantation, WT bone marrow was used to reconstitute irradiated WT or *St6gal1−/−* hosts. We observed reduced total IgG levels in *St6gal1−/−* chimeras at 6- and 8-weeks post-transplantation, though this difference disappeared by 10 weeks (Figure 1G). *St6gal1−/−* recipients also exhibited a compromised primary antigen-specific IgG response to NP-ovalbumin immunization when compared to *St6gal1*+*/*+ recipients, even 12 weeks post-transplantation (Figure 1H). Importantly, these observations could not be attributed to altered IgG half-life, which was unchanged by host ST6GAL1 expression in exogenous administration experiments (Supplementary Figure S2).

### Hepatic ST6GAL1 Modifies Mature B Cell Sialylation and Function

**FIGURE 2:**
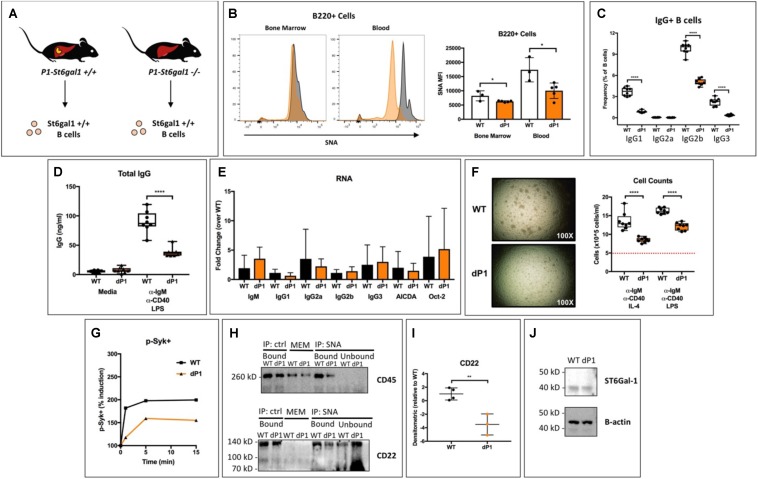
Hepatic ST6Gal-1 modulates mature B cell sialylation, proliferation, and IgG production. **(A)** Bone marrow IgD + mature B cells were isolated from WT and dP1 mice, which lack the P1 promoter for *St6gal1* operant within hepatocytes. **(B)** SNA reactivity of bone marrow and peripheral blood B220 + B cells by histogram (left) and MFI (right, *n* = 3–4). Mature B cells were grown at equal numbers for 72 h in media or with indicated stimulus (*n* = 6–8). Frequency of **(C)** IgG + B cells and **(D)** total IgG in cell-free conditioned medium were quantified. **(E)** RT-qPCR quantification of IgG germline transcripts (GLT), AICDA, and Oct-2 at the end of culture period (*n* = 5). **(F)** Altered cellular proliferation after stimulation of dP1 mature B cells for 3 days (dashed red line indicates seeded cell concentration). **(G)** Altered Syk phosphorylation in dP1 B cells after BCR activation. **(H)** Sialylated cell surface proteins were immunoprecipitated from the membrane fraction of resting WT and dP1 IgD + B cells using SNA-conjugated agarose beads. Total membrane (MEM) proteins, bound and unbound fractions for both SNA-agarose and agarose control were immunoblotted for indicated proteins. **(I)** Quantification of SNA-immunoprecipitated CD22 from *n* = 3–4 individual mice. **(J)** Immunoblot of cytosolic ST6GAL1. *p < 0.05, **p < 0.01, ****p < 0.0001.

Blood levels of ST6GAL1 peak late during the acute phase response to inflammation (3, 4). Four or more spatially and operationally distinct promoter regions drive transcription of the *St6gal1* gene in mice and humans (34–36). Although both hematopoietic and hepatic expression contribute to extracellular ST6GAL1 enzyme levels at steady-state, the 4-fold induction of expression that occurs during inflammation results from transcriptional activity at the P1 promoter of adult liver, which is not active within B-lineage cells (5, 17, 31, 35, 37). Our results from Figure 1 indicate that ST6GAL1 alters the production of IgG in mature B cells by a cell non-autonomous mechanism. In order to gain further clarity into whether liver-derived ST6GAL1 was mediating this effect, we analyzed the *dP1* mouse, which harbors a targeted deletion of the P1 promoter necessary for induction of ST6GAL1 in the liver (Figure 2A). B cells from WT and *dP1* mice are equally capable of endogenous ST6GAL1 expression and differ only in their exposure to circulating, environmental ST6GAL1. Surprisingly, *dP1* B220 + cells exhibited a significant reduction in cell surface sialylation compared to WT controls, with a larger difference noted in the peripheral, circulating population (Figure 2B). To understand if this was associated with any functional changes, bone marrow mature (IgD+) B cells of WT and *dP1* mice were isolated by magnetic separation, plated at identical cell numbers, and stimulated with anti-IgM and anti-CD40 antibodies, as well as LPS, to induce class switching (32). After 72 h, flow cytometry analysis indicated striking reductions in the frequency of class-switched IgG1+, IgG2b+, and IgG3+ B cells in *dP1* cultures (Figure 2C). Furthermore, stimulated *dP1* B cells secreted significantly less IgG compared to WT controls (Figure 2D). At the RNA level, primary inducers of class-switch recombination (*AICDA, Oct2*) and germline transcripts for IgG1, IgG2a, IgG2b, and IgG3 remained unchanged, suggesting isotype switching was unaltered by circulating liver-derived ST6GAL1 (Figure 2E). Rather, cell proliferation was significantly blunted in *dP1* cells, along with reduced phosphorylation of Syk after BCR stimulation (Figures 2F,G). These results suggest that lack of liver-derived ST6GAL1 compromises IgG production in B cells due to altered BCR signaling and antigen-induced proliferation, not an inability to undergo class-switch recombination.

The ability of α2,6-sialic acid conjugates to restrain CD22-mediated inhibition has been attributed to the formation of CD22 homomultimers and CD22-CD45 complexes (25, 38). To understand whether hepatic ST6GAL1 directly alters sialylation of B cell membrane proteins, we blotted immunoprecipitated SNA-reactive membrane proteins from WT and *dP1* mature B cells (SNA, *Sambucus nigra agglutinin*, recognizes α2,6-sialylated structures). Interestingly, we observed a reduction in α2,6-sialylated CD45 and CD22 in *dP1* B cells, despite equivalent cytosolic levels of ST6GAL1 protein within B cells of both genotypes (Figures 2H–J).

### Extrinsic Sialylation Augments B Cell Maturation and IgG Production *ex vivo*

**FIGURE 3:**
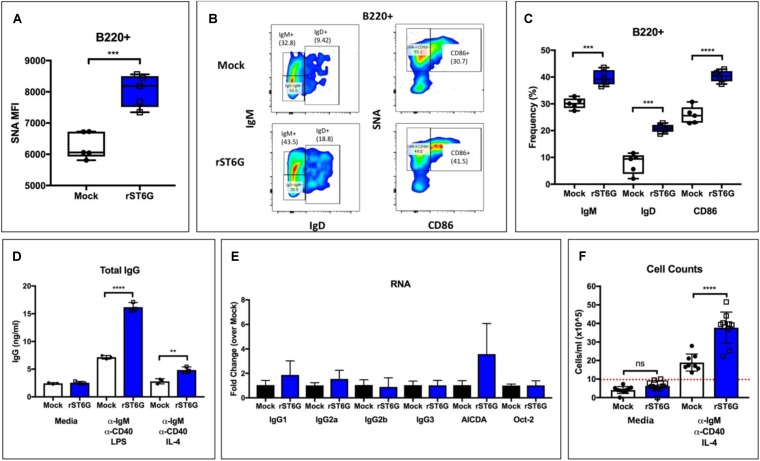
Extrinsic sialylation enhances development, proliferation, and IgG production in WT B cells. Wild-type bone marrow developing (B220 + /IgM–) B cells were isolated and matured for 3 days with BAFF, CMP-Sia and either recombinant rat ST6Gal-1 or mock buffer. **(A)** SNA reactivity of B cells after culture period (*n* = 5). **(B)** Representative staining of IgM, IgD, CD86, and SNA lectin. **(C)** Frequency of IgM+, IgD+, and CD86+ B cells after culture period (*n* = 5). **(D)** Treated cells were pelleted and washed twice with PBS, then cultured for 72 h in media or with indicated stimuli (*n* = 3). Total IgG in cell-free conditioned medium was quantitated (*n* = 3). **(E)** RT-qPCR quantification of immunoglobulin germline transcripts (GLT), AICDA, and Oct-2 was performed in LPS-treated samples (*n* = 3). **(F)** Total cell counts after stimulation (*n* = 8–10, dotted red line indicating starting concentration). **p < 0.01, ***p < 0.001, ****p < 0.0001.

Our observations point to an unexpected role for hepatic ST6GAL1 in IgG production by mature B cells. The altered sialylation of CD22 and CD45 on the B cell surface argue in favor of the direct modification of B cells by extracellular enzyme. Such extrinsic sialylation reactions can occur on a variety of leukocyte surfaces and are supported by charged sugar donor molecules from degranulating platelets (12, 13). We have previously reported low intrinsic expression of ST6GAL1 in immature B cells, which are specifically amenable to extrinsic sialylation by ST6GAL1 (30). To test whether extrinsic sialylation of immature B cells could boost IgG production to supraphysiologic levels, we isolated B220+/IgM*−* wild-type bone marrow B cells by magnetic separation and cultured them with recombinant BAFF, CMP-Sia donor substrate, and either recombinant ST6GAL1 (rST6G) or mock control. *Ex vivo* maturation of immature B cells into functionally responsive mature B cells by BAFF treatment has been previously reported (39). At 3 days, ST6GAL1 treated B cells had significantly increased α2,6-sialylation (Figure 3A), as well as cell surface IgM, IgD, and CD86 (Figures 3B,C). Next, the cultures were washed extensively to remove rST6G, CMP-Sia, and BAFF, and given fresh medium containing anti-IgM and anti-CD40 stimulating antibodies, along with either IL-4 or LPS for 3 days. Strikingly, B cells that were extrinsically sialylated secreted significantly more IgG in response to both IL-4 and LPS (Figure 3D). Consistent with the dP1 model, extrinsic sialylation did not significantly alter the abundance of transcripts necessary for the CSR process, including *AICDA, Oct*, and germline IgG subclasses (Figure 3E). Finally, cell counts indicated a significant increase in proliferation of rST6G-pretreated B cells after stimulation (∼2-fold higher), confirming that the enhanced IgG secretion resulted from robust antigen-induced proliferation of class-switched B cells, rather than a change in the process of CSR (Figure 3F).

### Elevation of Blood ST6GAL1 Alters CD22-Dependent B Cell Processes *in vivo*

Our data are consistent with a model wherein liver-derived ST6GAL1 extrinsically sialylates B cell surface CD22 and CD45, allowing for enhanced B cell receptor signaling, proliferation, and IgG production. These observations coincide with existing models of CD22 function, which propose a central role for the ligand-binding domain of CD22 in determining its membrane localization and inhibitory capacity (28, 40). However, it remains unclear if the mechanisms responsible for our observations are dependent on the expression of CD22. Moreover, since the extracellular nature of this pathway suggests that manipulation of blood ST6GAL1 may be a feasible treatment for hypogammaglobulinemia, we analyzed the effects of blood ST6GAL1 supplementation in CD22-sufficient and CD22-deficient mice.

**FIGURE 4:**
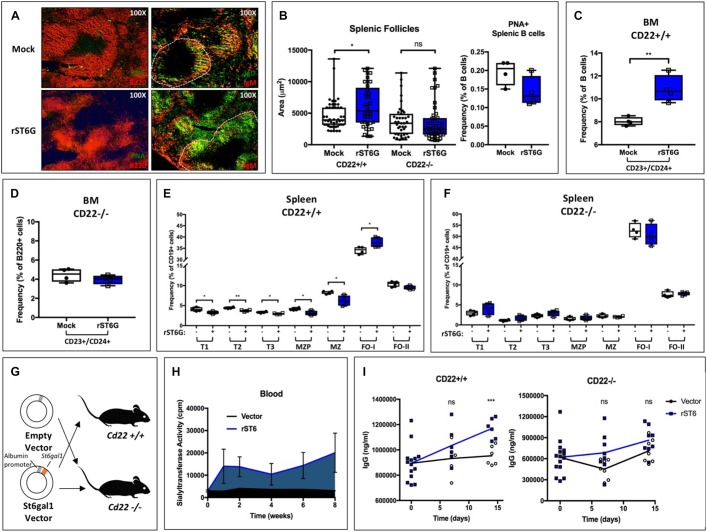
Supplementation of blood ST6GAL1 bolsters B cell development and blood IgG levels by a CD22-dependent mechanism. Recombinant rat ST6GAL1 (rST6G) or mock was intravenously administered into wild-type mice, along with CMP-Sia and BAFF. On day 4 post-injection, mice were sacrificed for analysis. **(A)** Representative images of spleens of rST6G-treated or mock control mice at day 4. In left panels, B220 (red) and peanut agglutinin (PNA, green) are shown. In right panels, IgM (red) and IgD (green) are shown, with a single follicle outlined (white, dashed). **(B)** Distribution of splenic follicle size in rST6G-treated WT and CD22*–*/*–* mice (*n* = 37–46, left). B cell activation was unchanged by treatment (*n* = 4, right). B cell populations were analyzed by flow cytometry in CD22+/+ and CD22*–*/*–* mice. **(C)** and **(D)** Frequency of bone marrow CD23+/CD24+ late transitional B cells is shown (*n* = 4). **(E)** and **(F)** Frequencies of splenic B cell subpopulations in rST6G-treated mice (*n* = 4). All comparisons in **(F)** are not statistically significant. **(G)** Schematic for hydrodynamic transfection of WT or CD22*–*/*–* mice with pLive plasmid containing rat *St6gal1* under the murine albumin promoter or empty vector control. **(H)** St6gal1-KO mice were subjected to hydrodynamic transfection. Blood sialyltransferase activity is shown up to 8 weeks post-transfection (*n* = 3). **(I)** Hydodynamic transfection was performed with CD22+/+ (WT) or CD22*–*/*–* mice, and total serum IgG quantified over 2 weeks (*n* = 6). *p < 0.05, **p < 0.01, ***p < 0.001.

First, we tested the short-term effects of a single intravenous bolus of rST6G (or mock control) on B cell development. All mice also received intravenous CMP-Sia sugar donor. In order to enhance B cell development, mice were administered concurrent recombinant murine BAFF, as has been reported elsewhere (41). After 4 days, mice were euthanized, and bone marrow and splenic B cells harvested for analysis. To visualize splenic architecture, germinal center B cells were stained using Peanut agglutinin (PNA) and B220 (Figure 4A, left). We also identified the follicular and marginal zone B cell populations using IgM and IgD (Figure 4A, right). Although B cell activation status was unaltered, treatment with rST6G increased IgD staining within follicles as well as overall follicle size. Treatment of CD22*−*/*−* mice with rST6G, however, failed to increase follicle size (Figure 4B). Flow cytometry analysis demonstrated that rST6G significantly increased bone marrow CD23+/CD24+ cells, a population roughly corresponding to BAFFR + late-transitional B cells previously described (Figure 4C) (42, 43). This population was not changed by rST6G treatment of CD22−/− mice (Figure 4D). Within the spleen, relative frequencies of immature (T1, T2, T3) and marginal zone lineage (MZP, MZ) B cells were reduced by rST6G, whereas the IgD + type I follicular B cell population was enlarged (Figure 4E). Treatment of CD22*−*/*−* mice did not reproduce these changes, underscoring the necessity for CD22 in the function of extrinsic ST6GAL1 *in vivo* (Figure 4F).

To test if prolonged elevation of blood ST6GAL1 can augment *in vivo* IgG production, we developed a model in which systemic ST6GAL1 levels are artificially elevated by hepatic overexpression. A plasmid encoding the secretory form of rat *St6gal1* under the albumin promoter was constructed, ensuring hepatocyte-restricted expression of the transgene. The expressed enzyme lacks the transmembrane domain and is thus uniformly secreted (Figure 4G). Hydrodynamic transfection of liver cells was achieved by intravenous injection of plasmid in 2 mL saline (44). In comparison to vector controls, *St6gal1*-KO mice receiving rat ST6GAL1 (rST6) plasmid exhibited a significant and prolonged increase in blood sialyltransferase activity up to at least 8 weeks post-transfection (Figure 4H). Strikingly, at 2 weeks post-transfection, rST6-treated mice exhibited higher total IgG levels (*p* < 0.001). This effect was abrogated in CD22−/− mice, wherein blood IgG titers did not significantly differ between treatment and control groups (Figure 4I). Collectively, our results demonstrate the utility of modulating blood ST6GAL1 levels for the alteration of CD22-dependent B cell ontogeny and IgG production.

## Discussion

B lymphocytes produce immunoglobulins that protect the host from extracellular pathogens throughout life. Disruption of B cell functions occurs in both primary immunodeficiency and the iatrogenic ablation of B cell precursors. After bone marrow transplantation, the delayed recovery of humoral immunity predisposes patients to recurrent infections with encapsulated bacteria (45). This humoral deficit is the result of both reduced B cell numbers and diminished B cell functions such as proliferation and antibody production (46–48). Treatment strategies focus on the passive transfer of antibodies or antigen-specific B cells, which can be prohibitively expensive and do not correct underlying functional defects (49, 50). This study demonstrates the importance of a B cell-independent glycosyltransferase in sustaining systemic IgG levels *in vivo*. Considering the central role for supporting hematopoietic cells in antigen presentation (e.g., dendritic cells, macrophages) and co-stimulation (e.g., T cells), as well as the potential for antigens themselves to harbor glycans, it is worth noting that total blood IgG levels may represent the final outcome of perturbed glycosylation in a number of cell types (51).

We present multiple, independent lines of observations implicating circulating ST6GAL1, rather than the sialyltransferase pool natively expressed within B cells, as prominent regulators of B cell development, proliferation and IgG production. First, ST6GAL1-competent hematopoietic compartment re-established in a ST6GAL1-null host have diminished B cell development and delayed IgG production (Figure 1). While much of the circulating ST6GAL1 originated from the liver, disabling the transcription promoter specific for liver *St6gal1* expression resulted in a similar presentation of diminished B cell proliferation and IgG production (Figure 2). Finally, supplementation of extracellular recombinant ST6GAL1, *in vitro* and *in vivo*, achieved the opposite effect, that of enhancing development, proliferation, an IgG production (Figures 3, 4). Our collective findings are most consistent with a novel axis of liver-humoral crosstalk mediated by secreted ST6GAL1, regulating B cell sensitivity to antigen engagement and capacity for immunoglobulin production.

Our data highlight an underappreciated role of the liver in the immune response, adding to its established function in regulating bone marrow platelet production by the secretion of thrombopoietin (52, 53). Though it is not known if variations in blood ST6GAL1 correlate with blood IgG titers in either transplant recipients or healthy controls, our data show that even the supplementation of ST6GAL1 to supraphysiologic levels can alter the development and function of healthy B cells. Furthermore, the extracellular nature of this pathway lends itself to direct therapeutic translation, as recombinant ST6GAL1 was sufficient to enhance BAFF-dependent development, and prolonged elevation of ST6GAL1 raised total serum IgG. Importantly, our findings here are consistent with a CD22-dependent mechanism, and are likely translatable to the human context, where CD22 is expressed and has conserved functions in B cell signaling (54). Although humoral immunodeficiencies due to the complete loss of B cell functions (e.g., X-linked agammaglobulinemia or Hyper-IgM syndrome) are unlikely to respond to extrinsic sialylation, conditions in which B cells develop but exhibit sub-optimal function (e.g., Common variable immunodeficiency) may yet be amenable to extrinsic ST6GAL1 therapy (55).

Protein sialylation is widely regarded as a means of intercellular communication between leukocytes and their environment. α2,6-sialylation of IgG, which is suppressed by pro-inflammatory IL-21 and IL-22, prevents myeloid cell activation by enabling binding to FcγRIIb, thereby ameliorating tissue damage in inflammatory arthritis (18–20). α2,6-sialic acid also has a direct function on the B cell surface through the engagement of CD22 (Siglec-2) and Siglec-G. In B2 cells, CD22’s intracellular domain suppresses signaling by the recruitment of SHP-1 phosphatases to the B cell receptor complex (26–28). The extracellular ligand-binding domain of CD22 and the acetylation of the sialic acid ligand are necessary for these effects (28, 56). Thus, engagement of CD22 with sialylated auto-antigens in *trans* is likely to promote tolerance (51, 57). Paradoxically, the primary function of α2,6-sialylation is in fact to restrain CD22-mediated inhibition, as global ST6GAL1 deficiency leads to B cell hypoactivity that is reversed in ST6GAL1/CD22 compound knockouts (23, 25, 58). Despite reported interactions with IgM and CD45, the majority of membrane CD22 homo-multimerizes in clathrin rafts by lectin-dependent interactions, sequestered away from the activated BCR complexes in lipid rafts (59, 60). Indeed, recent studies suggest that both CD22-CD22 and CD22-CD45 complexes improve BCR signaling (38, 61).

The maintenance of cell surface sialic acid is achieved by the combined actions of intrinsically expressed and extracellular sialyltransferases. Although the relative importance of either pathway remains to be elucidated, the balance is likely dynamic and reflective of changing physiologic states, as inflammatory stimuli alter the availability of both the enzymes and platelet-derived sugars necessary for extrinsic sialylation reactions (5, 12). Liver is the principal source of systemically circulating extracellular ST6GAL1. Deletion of the *St6gal1* transcription promoter uniquely utilized by hepatocytes resulted in dramatically decreased levels of blood-borne ST6GAL1 at baseline and especially during systemic inflammation (31). Other cell types can also perturb local ST6GAL1 levels and contribute to systemic levels as well. For example, bone marrow IgD + B cells and plasma cells secret significant amounts of active ST6GAL1 that can extrinsically sialylate hematopoietic progenitors to inhibit granulopoiesis in the marrow, and ST6GAL1 released from the B cells contributes to the overall systemic pool at baseline^7^.

The current report documents a role for the systemic pool of ST6GAL1 in mediating CD22-dependent signaling. While the *cis* ligands of CD22 are conventionally assumed to be generated cell-autonomously by the B cells, the data show that *cis* ligands of CD22 can also be generated cell non-autonomously by ST6GAL1 in circulation. The autonomy of B cells in their own sialylation is likely influenced by ontogeny, as the expression of endogenous ST6GAL1 in B cells varies significantly during development, with highest expression in splenic early transitional and activated germinal center B cells (30). Of particular interest, immature B cell populations, with low intrinsic ST6GAL1 expression, may represent a stage during which hepatic ST6GAL1 compensates for poorly sialylated B cell proteins, enabling systemic control over CD22 biology. It is thought that the availability of charged sugar donors is a major limitation for extracellular sialylation reactions. The source of the charged sugars driving the sialylation of B cells remains an open question. Although platelets have been shown to be relevant donors in the periphery, it is unclear if platelet progenitors (i.e., megakaryocytes) or necrotic cells can carry out similar functions in the marrow (11, 12, 62). Others have already observed a role for megakaryocytes in marrow B cell development starting at the pre-pro-B cell stage, consistent with this model (63).

Furthermore, global states of stress and homeostasis likely influence the balance of hepatic and lymphocytic ST6GAL1 production, dynamically altering the importance of these parallel pathways *in vivo* (64). Our data show that artificial elevation of blood ST6GAL1 activity profoundly enhanced CD22-dependent circulating IgG levels after 2 weeks. This raises the possibility that similar effects may be achieved by infusion of recombinant ST6GAL1, which we have already shown is sufficient to both upregulate follicular B cell development and mitigate neutrophilic airway inflammation (6).

The paradigm proposed here roughly parallels the growing understanding of extrinsic sialylation in the generation of glycans on soluble immunoglobulins, which are direct mediators of diverse effector functions and function independently of CD22 (65, 66). The ability of extrinsic sialylation by bloodborne ST6GAL1 to modify the sialylation of secreted IgG has been a topic of debate, as maintenance of sialic acid is critical to the effects of IVIG (67–69). However, though the extrinsic pathway has been demonstrated by therapeutic administration of recombinant enzyme in models of inflammatory disease, endogenous extracellular ST6GAL1 in healthy mice does not significantly alter the sialylation of administered IVIG (62, 70). Ultimately, the activation of extrinsic pathways of glycosylation may be limited by the physiologic availability of sugar donor molecules, which become increasingly available during time of stress and inflammation (12).

Generally, the defense against extracellular pathogens requires both microbicidal neutrophils and antigen-specific immunoglobulins (71). Although extracellular glycosyltransferases have been noted for decades, their relevance to physiology has been chronically overlooked (72–74). Nevertheless, a role for circulatory ST6GAL1 in immunity and hematopoiesis is emerging, with specific roles in the control of bone marrow neutrophil and eosinophil production, as well as the sialylation of plasma proteins (21, 22, 67, 75). The findings presented here extend the targets of extracellular ST6GAL1 to include the B cell lineage, demonstrating the importance of a systemic sialyltransferase to both the innate and adaptive branches of immunity. Collectively, findings by our group and others point to the ability of extrinsic sialylation to alter cellular sensitivity to exogenous ligands, including growth factors, cytokines, and antigens (14, 15, 17, 30). Temporally, blood ST6GAL1 is diminished early in inflammation and rises above baseline at a time coinciding with the resolution of acute inflammation (4, 6). In this context, extracellular ST6GAL1 likely functions to suppress excess inflammatory cell production while priming developing B cells for imminent antigen presentation, balancing the evolving needs for key leukocyte lineages during an immune response.

## Data Availability Statement

The raw data supporting the conclusion of this manuscript will be made available by the authors, without undue reservation, to any qualified researcher.

## Ethics Statement

The animal study was reviewed and approved by the Roswell Park Institutional Animal Care and Use Committee.

## Author Contributions

EI contributed to the conceptualization, methodology, investigation, formal analysis, writing (original draft preparation and writing), review, and editing. PP contributed to the conceptualization, methodology, and formal analysis. JL contributed to the conceptualization, resources, writing (review and editing), supervision, projection administration, and funding acquisition.

## Conflict of Interest

The authors declare that the research was conducted in the absence of any commercial or financial relationships that could be construed as a potential conflict of interest.
