# Supplementary material for: Blood-Borne ST6GAL1 Regulates Immunoglobulin Production in B Cells
Source: Front Immunol. 2020 Apr 23;11:617. doi: 10.3389/fimmu.2020.00617 (PMC7190976; doi:10.3389/fimmu.2020.00617)
Supplement: Supplementary file 1 [file Image_1.pdf]

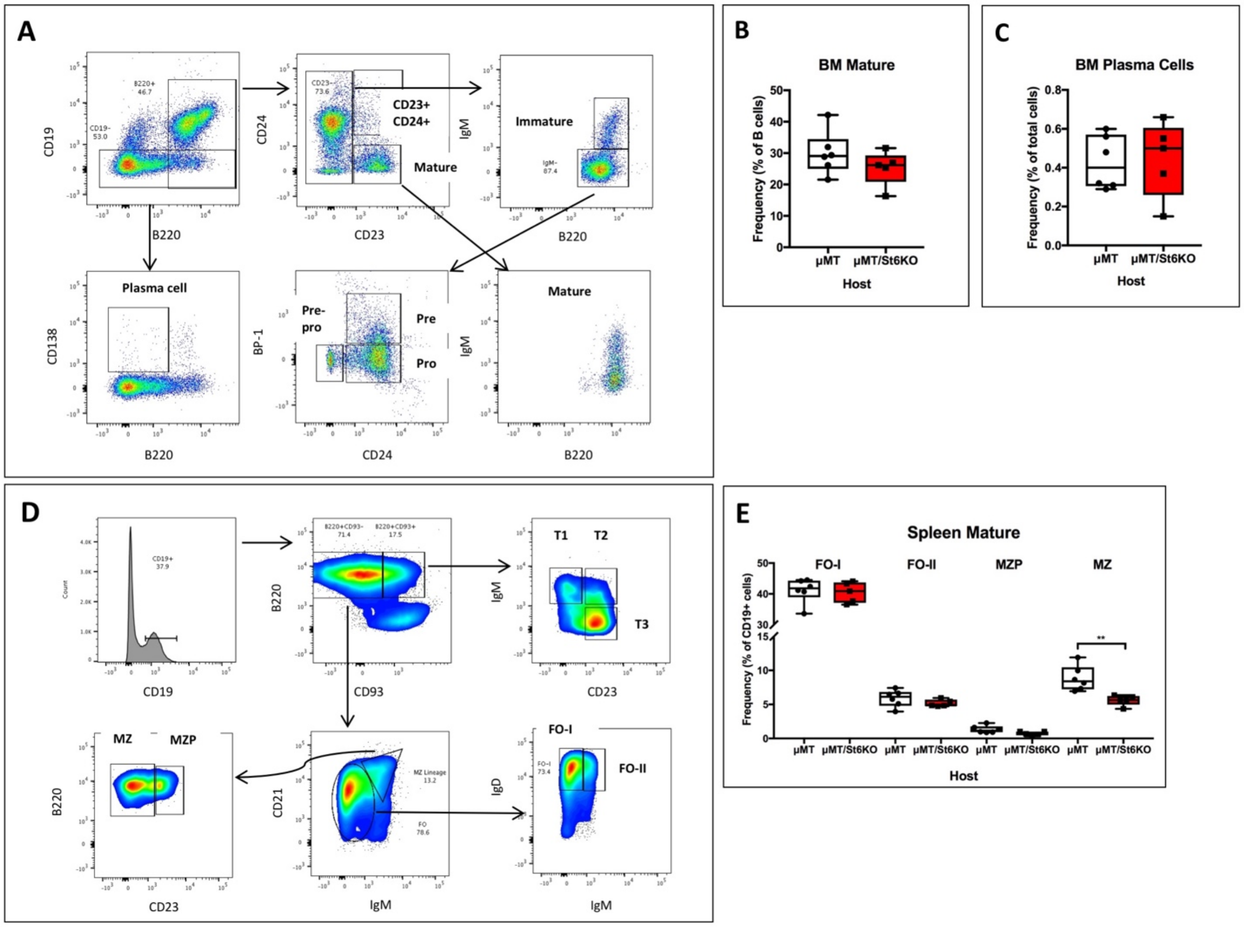

Supplementary Figure S1. Flow cytometry gating scheme and B cell reconstitution. (A) Gating of bone marrow B cell subpopulations. Quantification of bone marrow (B) mature B cells and (C) plasma cells in chimeras (n=5-6). (D) Gating of splenic B cell subpopulations. (E) Quantification of splenic B cell populations in chimeras (N=5-6). ** p<0.01
