# Supplementary material for: Blood-Borne ST6GAL1 Regulates Immunoglobulin Production in B Cells
Source: Front Immunol. 2020 Apr 23;11:617. doi: 10.3389/fimmu.2020.00617 (PMC7190976; doi:10.3389/fimmu.2020.00617)
Supplement: Supplementary file 2 [file Image_2.pdf]

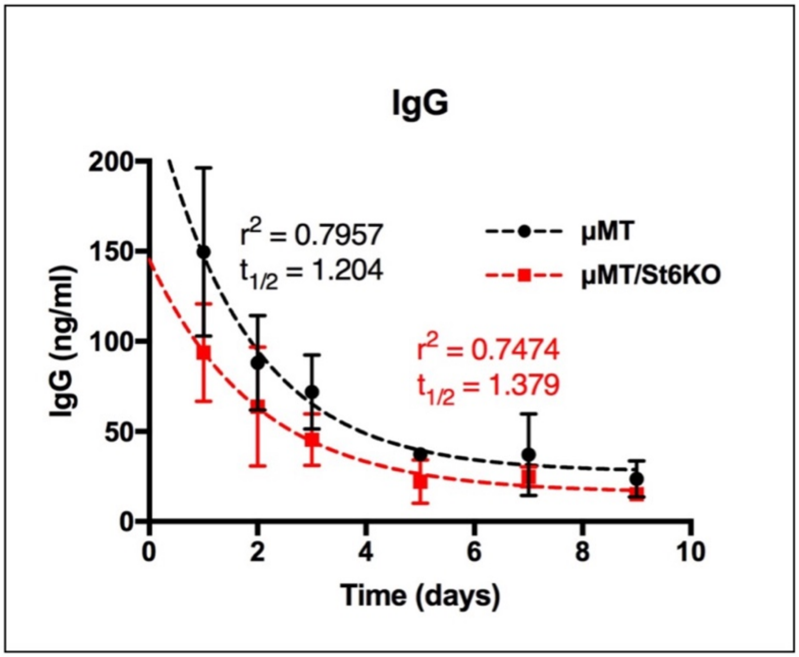

Supplementary Figure S2. Host ST6GAL1 does not alter IgG half-life. Exogenous polyclonal IgG was administered intraperitoneally to indicated mice on a B cell-deficient μMT background, and its clearance quantified in the serum (n=3). Best-fit exponential curves are shown.
